# Venous Thromboembolism following Elective Aesthetic Plastic Surgery: A Longitudinal Prospective Study in 1254 Patients

**DOI:** 10.1155/2014/565793

**Published:** 2014-10-09

**Authors:** Denis Souto Valente, Lauro Aita Carvalho, Rafaela Koehler Zanella, Sibelie Valente

**Affiliations:** Mãe de Deus Health System, Avenida Verissimo de Amaral 580-1704, 91360-470 Porto Alegre, RS, Brazil

## Abstract

*Background*. Venous thromboembolism (VTE) is a disorder with short-term mortality and long-term morbidity. Healthy patients submitted to elective aesthetic plastic surgeries (EAPS) have risk factors to develop VTE not well established yet. The objective of this study was to examine the incidence and risk factors for VTE in these patients. *Methods*. Longitudinal, prospective (minimum follow-up of 3 months), observational study. Comprehensive information on patient characteristics and surgeries performed was obtained. Preoperative, intraoperative, and postoperative risk factors were analyzed for their association with VTE. *Results*. A total of 1254 patients were included in the study. Postoperative VTE occurred in 17 (1,35%) of patients. VTE was more frequent in patients more than 40 years old (82.3%). Smoking, patients with 2 or 3 pregnancies, and hormone replacement therapy, and oral contraceptives use presents higher levels of VTE. In this study we have not found any correlation between liposuction, augmentation mammoplasty, mastopexy, and rhinoplasty as an isolated risk factor for VTE. *Conclusions*. The incidence of VTE in patients undergoing EAPS was 1.35%. Patients with more than 40 years of age, tobacco users, patients with 2 or more pregnancies, and hormone replacement therapy or oral contraceptives use presents higher levels of VTE.

## 1. Introduction

The significant increase in the popularity of aesthetic plastic surgeries in recent years has led to an increase in the number and types of complications associated with these procedures [1]. The postoperative occurrence of venous thromboembolism (VTE) is one of the most feared among these complications [2].

VTE, including deep venous thrombosis (DVT) and pulmonary embolus (PE), is a disorder with short-term mortality and long-term morbidity. The majority of PE deaths occur within hours of the embolic phenomenon, even when the event occurs in the hospital, often secondary to unrecognized DVT [3]. VTE carries the risk of sudden mortality but also notable morbidity, including the postthrombotic syndrome and pulmonary hypertension with progression to right heart failure [4].

Healthy patients submitted to elective aesthetic plastic surgeries (EAPS) have risk factors to develop VTE not well established yet, and there is no consensus regarding the use of VTE prophylaxis among them [5, 6]. In 2008, VTE risk stratification and prevention were identified by the Plastic Surgery Foundation Research Oversight Committee as the top patient safety research priority in this specialty [7]. Around 7% of plastic surgeons report a patient death from postoperative PE [8]. Unfortunately, we do not have, to date, a specific study of VET regarding only patients of aesthetic surgery.

In order to further understand the risk factors and incidence of VTE, the objective of this study was to examine the incidence and risk factors for VTE in patients undergoing EAPS.

## 2. Methods

This is a longitudinal, prospective (minimum follow-up of 3 months), observational study conducted at a private day hospital between January 2006 and June 2013 with consecutive patients of two plastic surgeons. All participants provided written and informed consent prior to study initiation and patient enrolment. This study is in accordance with the 2000 Edinburgh, Scotland Revision of the Declaration of Helsinki, applicable ICH guidelines, and Guidelines on Research Practice and is registered at the Brazilian Ministry of Health under the number 1111-1161-1277. Comprehensive information on patient characteristics, surgery performed, anesthesia type, surgery length, contraceptive or postmenopausal hormone replacement use, and comorbidities was obtained. A comprehensive summary of complications was also included for each patient. Preoperative, intraoperative, and postoperative risk factors were analyzed for their association with VTE. In addition, other complications associated with VTE were surveyed too.

The inclusion criteria were EAPS in ambulatory center, blood tests, and electrocardiogram with normal results before the surgery. The exclusion criteria were patients with any personal or familial history of VTE, blood dyscrasias, multiple surgeries, length of hospital stay greater than 24 h, and American Society of Anesthesiologists (ASA) physical status classification system 3 or higher.

Patients presenting clinical symptoms of VTE in the follow-up were screened by D-dimer assay. D-dimer testing is sensitive but not specific for identifying DVT; its use lowered the proportion of patients who needed ultrasonography. For patients with positive results, compression ultrasonography of the proximal veins in the symptomatic legs was conducted for suspected DVT and spiral computerized tomography for suspected PE.

All patients in this study did not receive chemoprophylaxis for VTE. We used intermittent pneumatic leg compression to enhance blood flow in the deep veins of the legs. This method is virtually free of side effects and is particularly useful in patients at high risk of bleeding, such as those undergoing plastic surgery. We also indicated early mobilization of patients as soon as possible after surgery.

In the statistical analysis, numerical variables were presented as mean (SD). The *t*-test was used to compare values of continuous variables between patients in whom VTE developed versus those in whom VTE did not develop whereas categorical variables were compared with the *χ* ^2^ test. For all analyses, a *P* < 0.05 was considered significant. Multivariate logistic regression analysis was used to identify the following risk factors of VTE. The odds ratio (OR) and 95% confidence interval (CI) for each independent variable are presented in the final regression model presented. For the statistical analysis, the statistical software SPSS 19.0 (SPSS Inc., Chicago, IL, USA) was used.

## 3. Results

A total of 1729 patients meet the initial inclusion criteria; among those 358 were excluded by the exclusion criteria, and 117 were lost to follow-up. So, 1254 patients were included in the final study with 3 or more months of follow-up. Of these patients, 864 (68.9%) were female and 390 (31.1%) male, and average age was 44.3 ± 16.2 years (range 15 to 72). Patients younger than 40 years constituted only approximately 29.7% of the total population. Postoperative VTE occurred in 17 (1.35%) of patients. Liposuction as the sole operative procedure was performed in 1007 (80.3%) of patients and mastopexy in 48 (3.8%); isolated breast augmentation was done in 132 (10.5%) and rhinoplasty in 67 (5.4%) patients. We did not find any difference in VTE incidence regarding the type of operative procedure.

In nearly 68.4% VTE occurred within the first 4 days after the surgery, with a mean delay of 3.8 ± 1.5 (median 3 days). Preoperative and intraoperative risk factors were presented in Table 1. VTE was more frequent in patients more than 40 years old (82.3%). Smoking, patients with 2 or 3 pregnancies, and hormone replacement therapy or oral contraceptives use presents higher levels of VTE. Preoperative diagnosis of lower limb varicose veins was not associated with a higher incidence of VTE (*P* = 0.237). Intraoperatively, patients who have surgeries lasting 2 or more hours, general anesthesia use, and liposuction procedures were not associated with an increased frequency of VTE. Multivariate analysis identified 4 independent factors as predictors of VTE as showed in Table 2.

## 4. Discussion

Many healthcare providers are under the false impression that this life-threatening illness is not a problem in their hospital or among their patients. While it is true that an individual plastic surgeon will normally see relatively few patients with this disease, it is clear that DVT is an important public health problem. The development of VTE and PE poses a small but significant risk for surgical patients and may result in death or debilitating consequences [4]. In our study VTE occurred in 1.35% of the patients. Most of the information used in VTE studies comes from reconstructive surgery. It is important to evaluate patients doing aesthetic procedures also. The results presented in this paper probably will stimulate further studies to allow comparisons.

Very little information exists on the incidence of VTE in the ambulatory surgery setting, especially amongst EAPS patients. However, the larger body of medical literature points to numerous intrinsic and transient risk factors that predispose a patient to VTE and PE [9, 10]. Patients with familial history of preoperative VTE, blood dyscrasias, multiple surgeries, length of hospital stay greater than 24 h, and ASA 3 or higher were excluded in our study because, according to the hospital policies, in these patients chemoprophylaxis for VTE must be performed.

These include a personal or family history of the disorders, venous insufficiency, chronic heart failure, obesity (body mass index 30 kg/m^2^), standing for more than 6 hours per day, history of more than two pregnancies, current pregnancy, violent effort or muscular trauma, deterioration in general condition, confinement to a bed and/or armchair, long-distance travel, infectious disease, use of general anesthesia during surgery, and performance of abdominoplasty with or without another procedure [11, 12].

In our study the use of oral contraceptive or hormone replacement therapy increases the risk of VTE. Literature shows that women with a current or recent history of contraceptive or postmenopausal hormone replacement use are also at increased risk for VTE and PE, especially if they have the factor V Leiden mutation; deficiencies in antithrombin, protein C, or protein S; or elevated levels of factor VIIIc. Not only have heritable mutations in coagulation factor V and/or prothrombin (i.e., G20210A) been associated with an increased risk of thromboembolism, particularly in white individuals, but also they have been shown to correlate with placental thrombosis during pregnancy, which itself is an acquired hypercoagulable state. The most definite of these pregnancy complications include stillbirth and preterm delivery and possibly recurrent miscarriage [13, 14].

In our study patients with 40 years of age or more showed a significant higher risk to develop VTE. A classic study showed that those patients are at an increased risk of VTE compared with younger patients, with the risk approximately doubling with each decade thereafter [15].

Smoking was also a risk factor to develop VET in our study. In a recent meta-analysis, involving approximately 4 million participants and more than 35,000 patients with VTE from 32 observational studies, a slightly increased risk of VTE for smokers was found compared with nonsmokers. The risk of developing VTE was greater for current smokers than for former smokers, and a dose-response relationship was found for daily smoking and pack-years smoked [16].

Much is said about liposuction risks; in this study we have not found any correlation between liposuction, augmentation mammoplasty, mastopexy, and rhinoplasty as an isolated risk factor for VTE. Despite the lack of reliable, comprehensive reporting of deaths and complications resulting from cosmetic surgeries, published data demonstrate that the risks associated with liposuction compare favorably with those from most general surgical procedures. A significant lack of literature documenting cosmetic breast surgery and rhinoplasty risks is observed, which should be of concern to both patients and physicians [17–20].

*Study Implications*. Having identified which patients are at risk of venous thromboembolism, the next choice for the physician is to select the most appropriate prophylactic measure for the patient's circumstances. The ideal primary prophylaxis should be effective, free from clinically important side effects, and well accepted by patients, nurses, and medical staff. It should be easy to administer, be relatively inexpensive, and require minimal monitoring.

Over the years, the available methods of thromboprophylaxis have been refined and improved to give maximum risk reduction for patients liable to suffer postoperative thrombosis. In principle, all prophylaxis is directed either at suppressing activation of blood coagulation or at increasing venous blood flow in the leg veins. There are two general types of prophylaxis—mechanical methods and pharmacological agents.

Ultimately, the decision to provide chemoprophylaxis should be made when the patient is initially admitted. Patients with age higher than 40 years old, who are smoking, with 2 or 3 pregnancies, and in hormone use at admission should be considered for early VTE prophylaxis using both mechanical and pharmacologic means.

## 5. Conclusion

The incidence of VTE in patients undergoing EAPS in this study was 1.35%. VTE was more frequent in patients more than 40 years old (82.3%). Smoking, patients with 2 or 3 pregnancies, and hormone replacement therapy or oral contraceptives use presents higher levels of VTE. In this study we have not found any correlation between liposuction, augmentation mammoplasty, mastopexy, and rhinoplasty as an isolated risk factor for VTE.

## Figures and Tables

**Table 1:** Risk factors of VTE studied.

Risk factor	Total	With VTE	Without VTE	*P* value
Age 41 or older	882/1254 (70.3%)	14/17 (82.3%)	743/882 (84.2%)	NS
Hormone therapy	714/1254 (56.9%)	15/17 (88.3%)	699/1237 (56.5%)	0.009
Smoking	504/1254 (40.2%)	5/17 (29.4%)	499/1237 (40.4%)	0.005
Varicose veins	132/1254 (10.5%)	1/17 (0.6%)	131/1237 (10.6%)	NS
Two or three pregnancies	67/1254 (5.3%)	2/17 (11.8%)	65/1237 (5.2%)	0.041
Surgery ≥ 2 h	155/1254 (12.4%)	1/17 (0.6%)	154/1237 (12.4%)	NS
General anesthesia	27/1254 (2.2%)	0/17 (0%)	27/1237 (2.2%)	NS
Liposuction	1007/1254 (80.3%)	13/17 (76.5%)	994/1237 (80.4%)	NS

**Table 2:** Multivariate logistic regression analysis of VTE risks factors.

Variable	Odds ratio	95% confidence interval	*P* value
Age > 40	0.504	0.335–0.756	0.000
Hormone therapy use	2.373	1.259–4.471	0.009
Nonsmoking	0.629	0.445–0.890	0.005
Two or three pregnancies	1.661	0.990–2.789	0.042

## References

[1] Valente DS, Zanella RK, Doncatto LF, Padoin AV (2014). Incidence and risk factors of striae distensae following breast augmentation surgery: a cohort study. *PLoS ONE*.

[2] Mustoe TA, Park E (2014). Evidence-based medicine: face lift. *Plastic and Reconstructive Surgery*.

[3] Guyatt GH, Eikelboom JW, Gould MK (2012). Approach to outcome measurement in the prevention of thrombosis in surgical and medical patients. Antithrombotic therapy and prevention of thrombosis, 9th ed: American College of Chest Physicians evidence-based clinical practice guidelines. *Chest*.

[4] Kearon C (2003). Natural history of venous thromboembolism. *Circulation*.

[5] Haeck PC, Swanson JA, Iverson RE, Lynch DJ (2009). Evidence-based patient safety advisory: patient assessment and prevention of pulmonary side effects in surgery. Part 2. Patient and procedural risk factors. *Plastic and reconstructive surgery*.

[6] The American Society of Plastic Surgeons http://www.plasticsurgery.org/Documents/medical-professionals/meetings-education/Complications/Pathways_to_Prevention_Guide.pdf.

[7] Pannucci CJ, Jaber RM, Zumsteg JM, Golgotiu V, Spratke LM, Wilkins EG (2011). Changing practice: implementation of a venous thromboembolism prophylaxis protocol at an academic medical center. *Plastic and Reconstructive Surgery*.

[8] Clavijo-Alvarez JA, Pannucci CJ, Oppenheimer AJ, Wilkins EG, Rubin JP (2011). Prevention of venous thromboembolism in body contouring surgery: a national survey of 596 ASPS surgeons. *Annals of Plastic Surgery*.

[9] Gould MK, Garcia DA, Wren SM (2012). Prevention of VTE in nonorthopedic surgical patients: Antithrombotic Therapy and Prevention of Thrombosis, 9th ed: American College of Chest Physicians Evidence-Based Clinical Practice Guidelines. *Chest*.

[10] Venturi ML, Davison SP, Caprini JA (2009). Prevention of venous thromboembolism in the plastic surgery patient: current guidelines and recommendations. *Aesthetic Surgery Journal*.

[11] Stevens WG, Vath SD, Stoker DA (2004). ‘Extreme’ cosmetic surgery: a retrospective study of morbidity in patients undergoing combined procedures. *Aesthetic Surgery Journal*.

[12] Tran BH, JoAnna Nguyen T, Hwang BH (2013). Risk factors associated with venous thromboembolism in 49,028 mastectomy patients. *Breast*.

[13] Pabinger I, Ay C (2009). Biomarkers and venous thromboembolism. *Arteriosclerosis, Thrombosis, and Vascular Biology*.

[14] James AH (2009). Venous thromboembolism in pregnancy. *Arteriosclerosis, Thrombosis, and Vascular Biology*.

[15] Anderson FA, Wheeler HB, Goldberg RJ (1991). A population-based perspective of the hospital incidence and case-fatality rates of deep vein thrombosis and pulmonary embolism: the Worcester DVT study. *Archives of Internal Medicine*.

[16] Cheng Y-J, Liu Z-H, Yao F-J (2013). Current and former smoking and risk for venous thromboembolism: a systematic review and meta-analysis. *PLoS Medicine*.

[17] Valente DS (2014). Preemptive analgesia with bupivacaine in reduction mammaplasty : a prospective, randomized, double-blind, placebo-controlled trial. *Plastic and Reconstructive Surgery*.

[18] Yoho RA, Romaine JJ, O'Neil D (2005). Review of the liposuction, abdominoplasty, and face-lift mortality and morbidity risk literature. *Dermatologic Surgery*.

[19] Campbell W, Pierson J, Cohen-Shohet R, Mast BA (2014). Maximizing chemoprophylaxis against venous thromboembolism in abdominoplasty patients with the use of preoperative heparin administration. *Annals of Plastic Surgery*.

[20] Miller TJ, Jeong HS, Davis K (2014). Evaluation of the American Society of Anesthesiologists physical status classification system in risk assessment for plastic and reconstructive surgery patients. *Aesthetic Surgery Journal*.

